# Commentary on Izquierdo (2024): Where next for exercise recommendations for healthy longevity in older adults?

**DOI:** 10.1016/j.jnha.2024.100421

**Published:** 2025-01-01

**Authors:** Adrian Bauman

**Affiliations:** School of Public Health, Faculty of Medicine and Health, Sydney University, Australia

The concept of healthy aging through exercise and physical activity has been described for several decades, first hypothesised in the 1970s [1,2]. More recent distillations have summarized the accumulated epidemiological and intervention evidence that supports approaches to healthy older adults [3,4] and are supported by World Health Organization 2020 recommendations (https://www.who.int/publications/i/item/9789240015128). Recently, recommendations have been developed using an international consensus process which emanated from the ICFSR (International Conference on Frailty and Sarcopenia Research) [5,6]. The most recent update (Izquierdo 2024) [7] provides comprehensive global recommendations for exercise and physical activity for enhancing longevity in older adults.

These recent recommendations are underpinned by an extensive review of the biology of aging, manifested as age-related declines in some physiological processes, in bone and muscle mass and declines in functional capacity. This extends the non-communicable disease prevention benefits of physical activity and distills evidence on the role of physical activity in offsetting age-related reductions in functional capacity and therefore, in improving independence and quality of life.

This has been described since 1980 as the ‘compression of morbidity’, whereby healthy lifestyles and exercise can postpone functional decline, resulting in healthier years added to the lives of older adults [8]; it was initially estimated that almost a decade more of healthy living could accrue, benefits greater than the prolongation of life among older adults [9].

This paper updates the ICFSR recommendations, further building on the evidence base from epidemiological studies, mechanistic studies that explore the biology of aging that may be reversed by exercise and physical activity and summarizing the intervention evidence for each proposed health benefit [7]. These comprehensive global guidelines describe the need for personalized and structured exercise regimens for older adults, especially focusing on the risks posed by chronic disease, frailty and sarcopenia. As programs should be individualized, they form part of the evolving discipline of precision medicine, tailoring exercise training to the needs and abilities of each person. Recommendations should include structured exercise programs supplemented by incidental lifestyle activity. Importantly, the recommendations strongly support multicomponent interventions, which extend beyond aerobic exercise and physical activity, and include progressive resistance training (PRT), as well as balance and flexibility training. The recommendations target both primary and secondary prevention for older adults, with the goal of improving quality of life for all elderly adults, including those with and without co-morbidities and existing disease. The target of these recommendations are health providers, and the recommendations should be integrated into routine clinical practice, could potentially reduce polypharmacy, and are likely to reduce health care costs. As well as improving management for people with chronic disease, the outcomes of comprehensive exercise and physical activity programs could decrease systemic inflammation, improve muscle function and functional capacity and possibly delay some cognitive decline.

The evidence base for action around physical activity and exercise for older adults is sufficient for population-level action and research translation, but this is an area least understood and poorly researched. This next stage is *research translation and delivery*, to reach more older adults across the population. Some of this work is in clinical settings and will need to be strongly linked to health system delivery mechanisms. One developed concept is ‘Exercise is Medicine’, a framework for primary care, and that defines exercise as a ‘vital sign’ in clinical assessment and management [10]. This has been considered in several countries, but its reach and dissemination has not been studied, and no population targets are established. For example, should clinicians target 20% or even 50% of all older adults in the community to be assessed and referred to exercise programs from primary care, and even greater proportions among those with diabetes or a history of falls? A challenge is the need for personalized exercise plans, and the benefits [and delivery challenges] of multicomponent interventions. These programs need to be delivered at scale in institutionalized settings (nursing and retirement homes), in free-living community settings and in hospital settings as part of geriatric care protocols. Research studies should test the best methods for implementing exercise protocols, and evaluation studies assess the reach of these scaled-up programs across the community. These principles are described in the WHO Toolkit for physical activity among older adults, with clear recommendations that sufficient resources, a delivery system and health system supports are essential [11]. One example is the scale-up of an exercise program for older adults across British Columbia in Canada, the ‘Choose to Move’ program, which has tested implementation, assessed local adaptation and monitored that effects at scale are sustained across diverse community settings [12,13].

Thus the evidence base is strong enough for recommending aerobic activities, resistance and balance training for all older adults to reduce frailty and improve functional capacity.

Multicomponent exercise programs will reduce health costs and could keep people independent and at home for several years more than those not exposed to such programs. The challenge remains to advocate for health system engagement and funding of such programs globally, and to develop delivery mechanisms in different countries and settings that still allow for flexible delivery of exercise programs tailored to the individual needs of older adults.

## Declaration of competing interest

The authors declare that they have no known competing financial interests or personal relationships that could have appeared to influence the work reported in this paper.
